# Infection with hepatitis B virus carrying novel pre-S/S gene mutations in female siblings vaccinated at birth: two case reports

**DOI:** 10.1186/1752-1947-4-190

**Published:** 2010-06-23

**Authors:** Ming-Wei Lai, Christopher S-H Yeh, Chau-Ting Yeh

**Affiliations:** 1 Division of Pediatric Gastroenterology, Chang Gung Children's Hospital, Taoyuan, Taiwan; 2 Graduate Institute of Clinical Medical Science, Chang Gung University College of Medicine, Taoyuan, Taiwan; 3 Liver Research Unit, Department of Gastroenterology, Chang Gung Memorial Hospital, Taipei, Taiwan

## Abstract

**Introduction:**

After the initiation of a mass hepatitis B vaccination program in Taiwan, the prevalence of hepatitis B virus infection has declined progressively. However, about 1 percent of the young generation, who received hepatitis B vaccination at birth, remain carriers. Infection with vaccine-escape hepatitis B virus mutants always occurs shortly after birth. Here, we report two female siblings in whom the infection occurred in their adolescence. This report raises the question of whether a booster for hepatitis B vaccination is needed.

**Case presentation:**

Two 19 and 14-year-old Taiwanese female siblings were born to a mother infected with hepatitis B virus and received a complete course of hepatitis B vaccination at birth. They remained negative for serum hepatitis B surface antigen and positive for serum anti-hepatitis B surface antibody throughout their childhood. However, both were infected with the hepatitis B virus in their adolescence. Hepatitis B virus DNA was extracted from serum samples from the mother and two siblings. Hepatitis B virus pre-S/S sequence was amplified by polymerase chain reaction followed by nucleotide sequencing. When compared with the sequence obtained from the mother, multiple amino acid substitutions located near or in the major hydrophilic region of the surface antigen were identified in the elder sister. Four of these mutations (sL97S, sL98S, sG102R, and sA159P) were novel. A novel in-frame deletion (14 amino acids deleted, pre-S 127-140) was found in the hepatitis B virus pre-S2 region in the younger sister.

**Conclusions:**

Despite having received hepatitis B vaccination at birth, hepatitis B virus infection can still occur in adolescence with the emergence of novel mutations in the pre-S/S gene. This is a rare event and, to the best of our knowledge, has not been previously reported.

## Introduction

After 22 years of nationwide hepatitis B vaccination in Taiwan, the hepatitis B virus (HBV) carrier rates in vaccinees have dropped from around 15 percent to below one percent [1-3]. The prevalence of serum hepatitis B core antibody has dropped from over 20 percent to below seven percent in freshmen and below three percent in younger people [3,4]. Despite the success of mass vaccination, breakthrough of HBV infection was reported in vaccinees, resulting in acute or chronic hepatitis [5-7]. The majority of HBV infections developed after immunization were caused by wild type viruses. However, up to 20 to 30 percent of breakthrough infections were proved to be caused by surface gene mutants, especially those with mutations located at the "*a*" determinant [8,9]. In 1990, a child developing protective antibody level after hepatitis B immunization was found to carry mutant HBV with a surface gene mutation at position 587 (guanine to adenosine), resulting in a change of glycine to arginine at amino acid 145 of the major surface antigen [10]. Subsequently, escape mutants at or outside the "*a*" determinant were reported in different countries [6,9,11]. Typically, these mutants emerged in those who had received either HBV vaccines or immunoglobulin [12]. However, they also developed in cases receiving anti-viral therapy or spontaneously occurred in chronic carriers [13-17]. These mutants not only escaped from host immunity, but also escaped from common diagnostic assays, posing a risk of spread through blood donation or horizontal transmission [12,18-20]. The long-term outcomes in patients with vaccine escape mutants have not been clearly defined, although such mutants were found in patients with hepatocellular carcinoma [20].

Here we report two female siblings, born to a carrier mother, positive for hepatitis B surface and e antigens (HBsAg and HBeAg). They both received hepatitis B vaccination at birth and were negative for HBsAg at eight years of age. However, HBV infection occurred in their adolescence. Pre-S/S gene mutants were identified in their serum samples.

## Case presentation

A 46-year-old carrier mother, positive for HBsAg and HBeAg, was regularly followed at our liver clinic since 2004. In June 2007, she brought her two daughters, aged 19 and 14 years old, to our out-patient clinic for examination of their serum markers for HBV. All three patients were Taiwanese. Owing to the universal vaccination program for HBV in Taiwan, the two sisters received a recombinant hepatitis B vaccination (Engerix-B; GlaxoSmithKline Biologicals, Rixensart, Belgium) at birth. They were both found to be positive for antibody against hepatitis B surface antigen (anti-HBs) and negative for HBsAg when they were eight years old. No symptoms related to hepatitis were noticed in the previous few years. Unfortunately, viral marker survey at our clinic (June 2007) showed that they were both positive for HBsAg and negative for anti-HBs. The elder daughter was positive for HBeAg and negative for antibody against hepatitis B e antigen (anti-HBe), while the younger one was the opposite. HBsAg and anti-HBs were evaluated by commercial radioimmunoassays (AusriaII and Ausab; Abbott Laboratories, North Chicago, IL). HBeAg and anti-HBe were also evaluated by radioimmunoassay (HBe RIA kit; Abbott Laboratories, North Chicago, IL). HBV-DNA level was 1.0×10^7 ^copies/mL in the elder sister and 1.9×10^8 ^copies/mL in the younger sister. HBV-DNA was quantified by Cobas Taqman HBV assay (Roche Molecular Systems, Branchburg, NJ). Serum alanine aminotransferase level was 132 U/mL in the elder sister and 44 U/mL in the younger one. Sequence analysis for HBV pre-S/S genes of the mother and her daughters was undertaken to determine if mutant strains were present. The younger sister subsequently received anti-viral therapy using entecavir 0.5 mg per day (Bristol-Myers Squibb, Princeton, NJ). The alanine aminotransferase level was normalized and HBV-DNA was suppressed to < 50 copies/mL one year later.

To extract HBV-DNA, serum (100 μL) was mixed with 300 μL of buffer (13.3 mM Tris HCl [pH 8.0], 6.7 mM ethylenediamine tetra-acetic acid, 0.67% sodium dodecylsulfate, and 150 μg/mL proteinase K) and incubated at 65°C for three hours. After phenol-chloroform extraction, DNA was subjected to polymerase chain reaction (PCR). The primers were P1, TTGGGAACAAGAGCTACAGC ATGG (nt. 2837-2860 sense) and P2, GCCTGTTAACAGGAAGT TTTCTAA (nt. 950-973, anti-sense). A serum sample obtained from a normal subject and an aliquot of water were included as negative controls. Direct sequence analysis was performed using an automatic DNA sequencer (CEQ 2000; Beckman Instruments, Fullerton, CA, USA).

**Figure 1:**
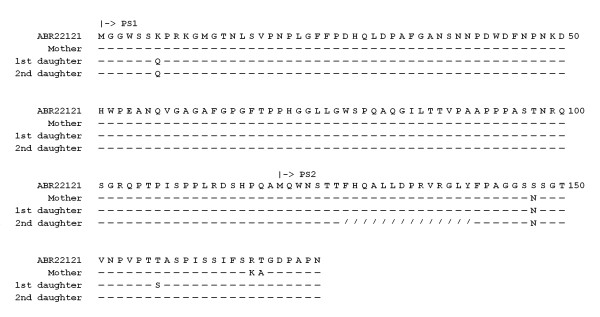
**Conceptually interpreted amino acid sequences of the pre-S gene of the mother and two daughters**. The sequences were compared with a reference hepatitis B virus sequence (genotype C, serotype adw, Genbank accession number ABR22121, at the top row). Amino acid residues identical to the reference sequence were represented by short lines. Deletions are represented by slashes. PS1, the initiation codon of the large surface protein; PS2, the initiation codon of the middle surface protein.

**Figure 2:**
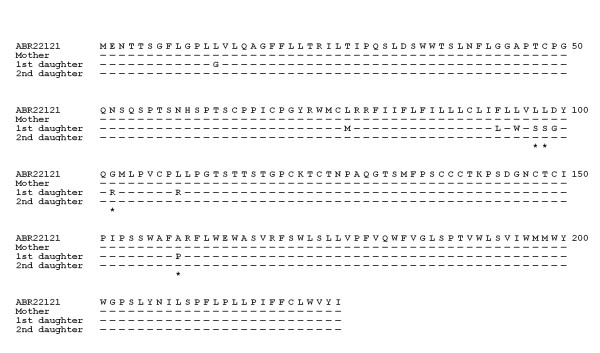
**Conceptually interpreted amino acid sequences of the S gene of the mother and two daughters**. The sequences were compared with the same reference sequence (see legend of Figure 1). Novel mutations were marked by asterisks.

After conceptual translation of the pre-S/S nucleotide sequences, the amino acid sequences were compared with those in GenBank using NCBI BLAST program. The sequences are shown in Figure 1 (pre-S region) and Figure 2 (S region). An HBV sequence was retrieved from GenBank and listed as a reference (genotype C, *adw*, Accession No. ABR22121). Amino acid substitutions that had not been reported in GenBank were considered novel.

It was discovered that the mother had a novel mutation at the pre-S2 region (psT168A), while the amino acid sequence in the S region was identical to that of the reference sequence. In the elder daughter, several amino acid substitutions near or in the major hydrophilic region outside the "*a*" determinant were found. Of these substitutions, four (sL97S, sL98S, sG102R, and sA159P) were novel. In the younger daughter, no novel amino acid substitution could be found in the S region. However, a short stretch of in-frame deletions (14 amino acid, a.a. 127 to 140) was found in the pre-S2 region.

## Discussion

In this report, the sisters had received hepatitis B vaccination at birth and successfully developed host immunity in childhood. However, they were subsequently infected by HBV in their adolescence, presumably due to decreased antibody titer resulting in inadequate protection. Compared with the non-immunized mother, the two vaccinated daughters harbored various mutations in the pre-S2 and S regions of hepatitis B envelop proteins. Although not in the commonly reported "*a*" determinant (a.a. 124-147), multiple mutations found in the elder sister were located near or in the major hydrophilic region (a.a. 99-170). The "*a*" determinant together with its surrounding areas is the major target for neutralizing antibody generated following vaccination. Therefore, the identified mutations were expected to alter the conformation of the surface protein, allowing for escape from vaccine-induced immunity. Notably, all three patients carried the sT126N mutation (Figure 2). This substitution, in combination with other mutations, has been reported in a surface antigen-negative HBV carrier [21].

The younger sister harbored a unique internal deletion in the pre-S2 region, not yet reported in the hepatitis B vaccinees. Tai *et al*. discovered frequent internal deletions of the pre-S2 region (pre-S, a.a. 120 to 140) in tumor parts of hepatocellular carcinoma, which usually occurred after several decades of chronic HBV infection [22]. The pre-S2 region contains several overlapping B and T cell epitopes, which invoke protective antibodies in chimpanzee and human hosts vaccinated with pre-S2-containing vaccines. However, it is noteworthy that the HBV vaccine they received (Engerix-B) does not contain pre-S proteins. Hence, the pre-S deletion is not likely to be selected by the antibodies generated by vaccination. At present, it is not clear why pre-S2 deletion can occur at such a young age.

Previously, vaccine-escape mutants were mostly discovered during patients' childhood when anti-HBs antibodies were still positive. In this report, we described a different group of "vaccine-escape" mutants. When patients who received hepatitis B vaccination at birth enter adolescence, the antibody titers decrease. In these patients, HBV infection could occur under inadequate host immunity. However, from our sequencing data, it is likely that the host immunity still play a role in selecting viral mutants, albeit insufficient to protect the host from HBV infection. Eventually, anti-HBs antibody was completely lost and the patients were infected with HBV. This study raises the question whether booster of hepatitis B vaccination in adolescents should be given provided that close contact with HBV carriers is not avoidable. However, this report is a rare event and more data are needed before a universal recommendation can be reached.

## Conclusions

We discovered two female siblings who had received hepatitis B vaccination at birth and successfully developed host immunity. However, they were subsequently infected by HBV in their adolescence. Sequence analysis revealed multiple novel mutations in the S gene near the "*a*" determinant region in the elder sister and a novel pre-S2 deletion in the younger sister.

## Consent

Written informed consent was obtained from the mother on behalf of both herself and her 14-year-old daughter and another consent was obtained from the 19-year-old daughter for publication of this case report and any accompanying images. A copy of the written consent is available for review by the Editor-in-Chief of this journal.

## Competing interests

The authors declare that they have no competing interests.

## Authors' contributions

MWL, CSHY and CTY analyzed and interpreted the sequence data. MWL and CSHY performed the DNA extraction and PCR. CTY designed the experiments. MWL and CTY were major contributors in writing the manuscript. All authors read and approved the final manuscript.
